# Characterization and analysis of long non-coding rna (lncRNA) in *In Vitro*- and *Ex Vivo*-derived cardiac progenitor cells

**DOI:** 10.1371/journal.pone.0180096

**Published:** 2017-06-22

**Authors:** Baron Arnone, Jake Y. Chen, Gangjian Qin

**Affiliations:** 1 Department of Biomedical Engineering, School of Medicine & School of Engineering, UAB, Birmingham, AL, United States of America; 2 Informatics Institute, School of Medicine, UAB, Birmingham, AL, United States of America; University of Illinois at Chicago, UNITED STATES

## Abstract

Recent advancements in cell-based therapies for the treatment of cardiovascular disease (CVD) show continuing promise for the use of transplanted stem and cardiac progenitor cells (CPCs) to promote cardiac restitution. However, a detailed understanding of the molecular mechanisms that control the development of these cells remains incomplete and is critical for optimizing their use in such therapy. Long non-coding (lnc) RNA has recently emerged as a crucial class of regulatory molecules involved in directing a variety of critical biological processes including development, homeostasis and disease. As such, a rising body of evidence suggests that they also play key regulatory roles in CPC development, though many questions remain regarding the expression landscape and specific identity of lncRNA involved in this process. To address this, we performed whole transcriptome sequencing of two murine CPC populations–Nkx2-5 EmGFP reporter-sorted embryonic stem (ES) cell-derived and *ex vivo*, cardiosphere-derived–in an effort to characterize their lncRNA profiles and potentially identify novel CPC regulators. The resulting sequencing data revealed an enrichment in both CPC populations for a panel of previously-identified lncRNA genes associated with cardiac differentiation. Additionally, a total of 1,678 differentially expressed and as-of-yet unannotated, putative lncRNA genes were found to be enriched for in the two CPC populations relative to undifferentiated ES cells.

## Introduction

Cardiovascular disease (CVD) is the leading cause of morbidity and mortality both in the US and worldwide [1]. The pathological endpoint for CVD is heart failure, which is characterized by significant loss of the myocardium due to acute or chronic cardiac injury, particularly myocardial infarction (MI), leading to a progressive reduction in cardiac function and, ultimately, death. Though multiple animal [2–4] and human [5, 6] studies have provided clear evidence for myocardial cell renewal throughout adulthood, this renewal capacity is vastly insufficient for the purposes of restoring cardiac mass and function for patients with chronic heart failure.

Over the past decade, cell-based transplantation has emerged as a promising therapeutic strategy for the treatment of patients with CVD [7, 8]. Preclinical animal studies have demonstrated the cardioregenerative potential of a variety of pluripotent cell types including embryonic (ESC) and induced pluripotent (iPS) stem cells–ESC and iPS, respectively–and those obtained from adult tissues including cardiosphere-derived cardiac progenitor cells and bone marrow (BM)-derived mesenchymal stromal cells–CPCs and MSCs, respectively. These findings have, in turn, led to an array of human clinical trials in CVD patient cohorts, though evidence demonstrating lasting therapeutic effectiveness in these trials remains inconclusive [9]. This is due in large part to an incomplete understanding of the specific cellular and molecular mechanisms by which these transplanted cells promote cardiac regeneration and improve functional restitution. The current conceptual paradigm has shifted away from a focus on transdifferentiation of transplanted cells and toward a model that emphasizes the secretory, paracrine factors that such cells produce which ultimately drive processes required for restorative cardiac remodeling including neovascularization, cardiomyogenesis and immunomodulation [10]. As such, a more detailed characterization of the molecular mechanisms that govern the development of and secretory profiles produced by stem and adult cardiac progenitor cells is crucial for maximizing future efficacy of cell-based therapies for CVD.

Long non-coding (lnc) RNA are a newly-emerging class of regulatory molecules that continue to gain recognition for their importance in a variety of critical biological processes including pluripotency, cell fate determination, homeostasis and disease [11]. As such, a growing body of evidence has demonstrated the significant role that lncRNA also play in regulating these processes within the heart [12, 13] and much of what is known has been discovered using *in vitro*, stem cell-based differentiation models to characterize the lncRNA transcription profiles of cardiac lineage-committed cells. Development-associated lncRNAs such as *Braveheart*, *Fendrr* and *Carmen* have been shown to be important for promoting cardiac lineage specification, partly through their interaction with chromatin remodeling complexes such as PRC2 which in turn target cardiac-specific transcription factor gene expression [14–16]. Examples of disease-associated lncRNAs include *Mhrt* which is a lncRNA that targets the chromatin modifying complex protein Brg1 and whose reduced expression is associated with cardiomyopathy [17], while MIAT (*m*yocardial *i*nfarction-*a*ssociated *t*ranscript) encodes for a 9 kilobase (Kb) transcript that contains 6 single nucleotide polymorphisms (SNPs), one of which is associated with increased levels of the *Miat* transcript levels and higher morbidity outcomes in MI patients [18]. Though the identity, molecular mechanism(s) and/or function of several cardiac-associated lncRNAs have been investigated, a more comprehensive characterization and analysis of the cardiac lncRNA transcriptomic landscape remains to be achieved, particularly as it relates to lncRNA that may be involved in stem and adult CPC development.

To that end, we performed whole transcriptome sequencing of RNA isolated from two murine CPC populations–one derived from cardiac-differentiated, Nkx2-5-EmGFP-sorted ESCs and the other from *ex vivo*, cardiosphere-derived c-kit+Lin- (CLK) cells–and assessed the differential expression of unannotated, putative lncRNA candidate transcripts in these cells relative to undifferentiated ESCs. A comparison of the sequencing data sets to that of a panel of 195 lncRNAs previously-identified in a similar cardiac development screen revealed a modest degree of overlap, with 108 lncRNA genes showing differential expression in the two CPC populations relative to control ESCs. Out of a total 1,678 unannotated genes that were found to be differentially expressed within the two CPC populations, 15 were commonly upregulated while 124 were commonly downregulated. Interestingly, several of these candidate lncRNA transcripts mapped to genomic regions proximal to protein-coding genes known to be involved in cardiac differentiation, indicating the potential for a *cis*-acting regulatory relationship similar to that displayed by other lncRNA. Further validation of these unannotated transcripts could lead to the identification of novel lncRNAs important for cardiac differentiation and provide new molecular targets for optimizing CPC development and advancing cell-based therapies in CVD.

## Materials and methods

### Cell culture and cardiac progenitor cell generation

The transgenic Nkx2-5 EmGFP mouse ES cell reporter line (129/OlaHsd background) was obtained from the Mutant Mouse Regional Resource Center (MMRRC; Stock# 030473-UCD). Cells were cultured and expanded in DMEM (4.5g/L glucose, L-glutamine and Sodium Pyruvate) containing 15% FBS (Atlanta Biologicals, S12450), 50uM BME, 1mM MEM and penicillin-streptomycin, supplemented with 10ng/mL LIF (Millipore, LIF2010) in order to maintain pluripotency. Embryoid body (EB) generation and cardiac lineage differentiation was carried out by plating 6x10^5^ cells/well in ultra-low attachment 6-well plates (VWR, 29443–030), feeding once every other day. Differentiation media consisted of IMDM (L-glutamine and 25mM HEPES) supplemented with 15% FBS, 200ug/mL Transferrin (Roche, 652202), 0.5mM ascorbic acid (Sigma, A4544), 0.45mM monothioglycerol (Sigma, M6145) and penicillin-streptomycin. Non-transgenic mouse ES cells (C57BL/6 background) were similarly cultured and used as a reporter control for EmGFP fluorescence. Cardiosphere-derived c-kit+Lin- (CLK) CPCs were generated and cultured as we previously described [19, 20].

### EB disaggregation, fluorescence-activated cell sorting and RNA isolation

A single cell suspension of the differentiating EBs was generated by using a combination of physical disruption and enzymatic digestion as previously described [21]. Briefly, EB aggregates from 3 wells of a 6-well ultra-low attachment plate were pooled and allowed to settle. Supernatant was removed and EBs were re-suspended in 0.5mL digestion solution of HEPES-buffered saline (HBS) containing 20% FBS, 10mg/mL Collagenase A (Roche, 10103578001), 10mg/mL Collagenase B (Roche, 11088807001), 10ug/mL DNase I (EMD Millipore, 260913), pH = 7.1. EBs were gently pipetted up and down 15X, transferred to a 1.7mL centrifuge tube and placed on a 37^°^C hotplate for one hour, pipetting up and down 15x every 15min. After one hour of digestion, cells were washed in FACS buffer (HBS with 20% FBS and 10ug/mL DNase I), stained with PI (Life Technologies, P3566) and analyzed on a BD LSR Fortessa flow cytometer. Live (PI-) EmGFP+ and EmGFP- cells were sorted into RNAprotect (Qiagen, 1038674) and RNA was isolated using RNeasy Plus Mini Kit (Qiagen, 74134). FACS data analysis was performed using FlowJo software (V8.8.7). For gene expression profiling of differentiating EBs not subjected to sorting, EB cultures were pooled as above and then RNA was directly isolated using RNeasy Plus Mini Kit.

### RT-PCR and gene expression analysis

cDNA was generated from whole cell lysate RNA using Multiscribe Reverse Transcriptase Kit (Applied Biosystems 4374966) and qPCR performed on an ABI 7900HT using Syber Green detection chemistry. Expression levels were normalized to β-actin. Expression data analysis was performed with Graphpad Prism software version 6.0e. Statistical significance was determined using one-way ANOVA and Bonferroni’s multiple comparisons test.

### RNA-Seq cDNA library generation and bioinformatics analysis pipeline

cDNA sequencing libraries were generated from total RNA using Illumina TrueSeq RNA Sample Preparation Kit v2 (Illumina, RS-122-2001). Libraries were sequenced in individual lanes for maximum depth on an Illumina HiSeq2500. Alignment and mapping of sequencing data was performed using Bowtie and Top Hat. A pseudo number of 1 was applied to each RPKM value for each corresponding sample gene and log_2_-transformed. A threshold of a fold-two increase or decrease in gene expression was used as a minimal inclusionary criterion for further downstream analysis. Unannotated reads were further selected on the basis of their mapped gene read lengths, whereby only putative genes whose transcript lengths spanned >200nt were assessed, as this reflects one of the central definitional features of lncRNAs and provides an important distinction with other ncRNA species such as miRNA.

## Results

### Nkx2.5 EmGFP CPC differentiation and characterization

**Fig 1:**
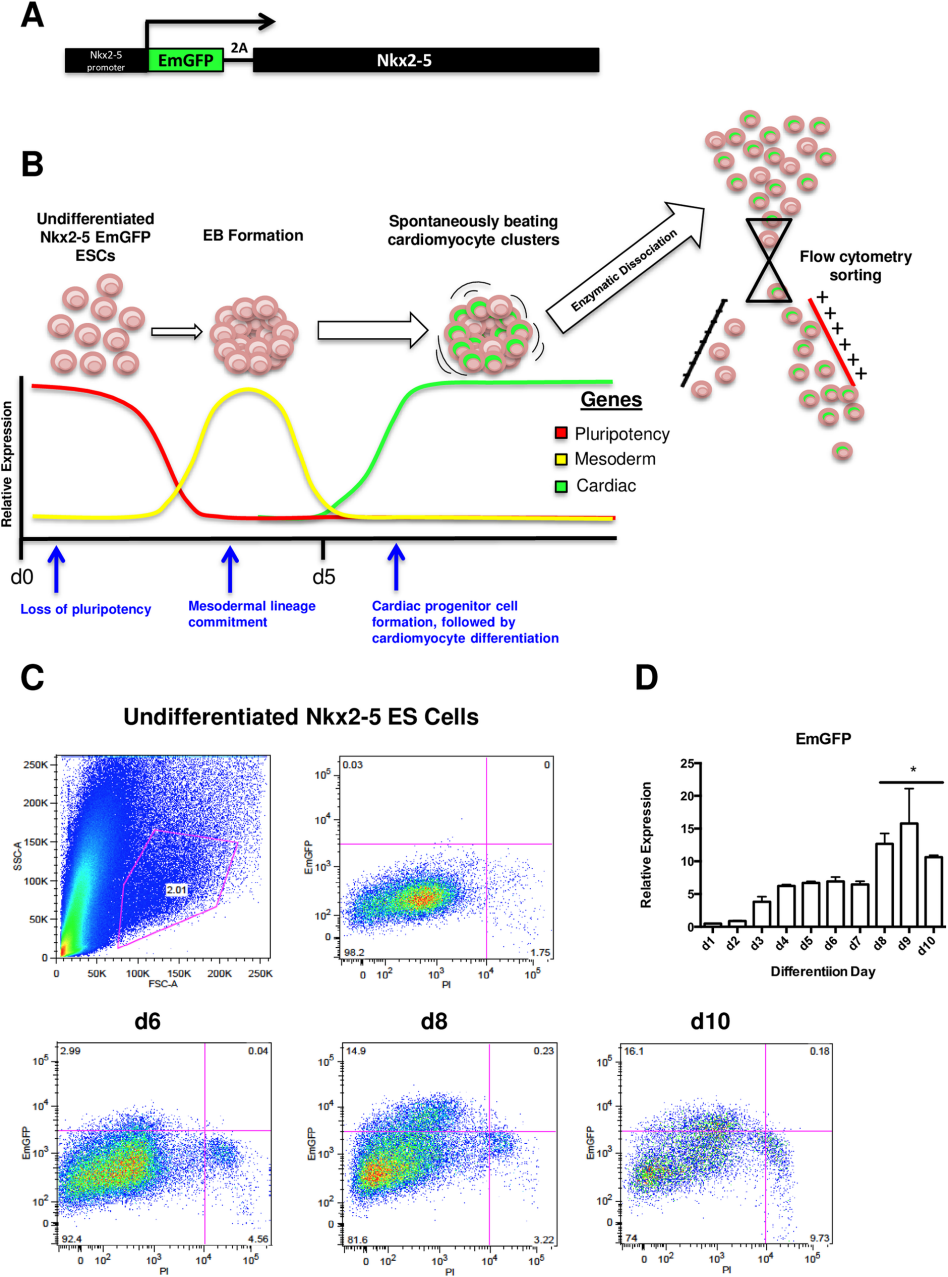
Schematic overview and FACS analysis of *in vitro*, murine Nkx2.5 EmGFP ES cell-based cardiac differentiation. **A)** schematic representation of homologously recombineered, 2A self-cleaving EmGFP reporter inserted immediately downstream of the endogenous Nkx2.5 promoter and immediately upstream of the Nkx2.5 gene in mouse ES cells, **B)** overview of gene expression trends for *in vitro* cardiac-directed differentiation, sorting and purification of Nkx2.5 EmGFP reporter ES cells, **C)** FACS analysis of Nkx2.5 EmGFP ES cells at d6, d8 and d10 of cardiac differentiation, **D)** EmGFP gene expression kinetics for non-sorted Nkx2.5 EmGFP ES cells throughout cardiac differentiation (* p<0.05, n = 3).

To establish a system for characterizing CPC-specific lncRNA expression, an *in vitro* model of cardiac differentiation was employed whereby mouse ES cells were cultured for 10 days under cardiogenic conditions [22] (**Fig 1B**). As an additional feature to enable CPC enrichment within this culturing system, ES cells contained a stably transduced EmGFP reporter under the control of the promoter for Nkx2-5 [23] (**Fig 1A**), an early developmental marker of CPCs and a critical transcription factor required for cardiac development [24], hereafter referred to as Nkx2-5 EmGFP cells. This reporter system enables the identification and selection of a multipotent EmGFP+ CPC population that, when purified, is capable of cardiomyocyte and vascular smooth muscle cell differentiation [25] and decidedly served as an ideal tool for isolating relatively pure populations of CPCs for the purposes of lncRNA expression profiling. A slight modification was made to the conventional *in vitro* approach to initiating cardiac differentiation from ES cells which typically utilizes a gravity-based hanging drop method to promote the formation of embryoid bodies (EBs) that are then plated onto an adherence matrix (e.g., gelatin). Alternatively, EB formation and differentiation was carried out in ultra-low adherence plates which, while still leading to spontaneous EB formation and subsequent cardiac differentiation [24], requires reduced protease-based digestion conditions and times for generating single cell suspensions for FACS analysis and provides higher yields of live differentiated cells.

We performed a combination of fluorescence-assisted cell sorting (FACS) and RT-PCR-based gene expression analyses throughout differentiation in order to ascertain the optimal time point at which Nkx2-5 EmGFP+ ES cell yields were the highest and transcriptional profiles were the most indicative of a cardiac lineage-committed cell population. EmGFP expression throughout differentiation showed a progressive increase over time, beginning at approximately day (d)3, though this increase was not statistically significant until d8 (**Fig 1D**). FACS analysis revealed that a higher percentage of EmGFP+ cells were present at d10 (**Fig 1C**), though the absolute number of live EmGFP+ cells was higher at d8. This was most likely due to less effective digestion of EBs at later time points, as the more differentiated cells secrete and deposit greater amounts of extracellular matrix proteins which ultimately inhibit their disaggregation (data not shown).

**Fig 2:**
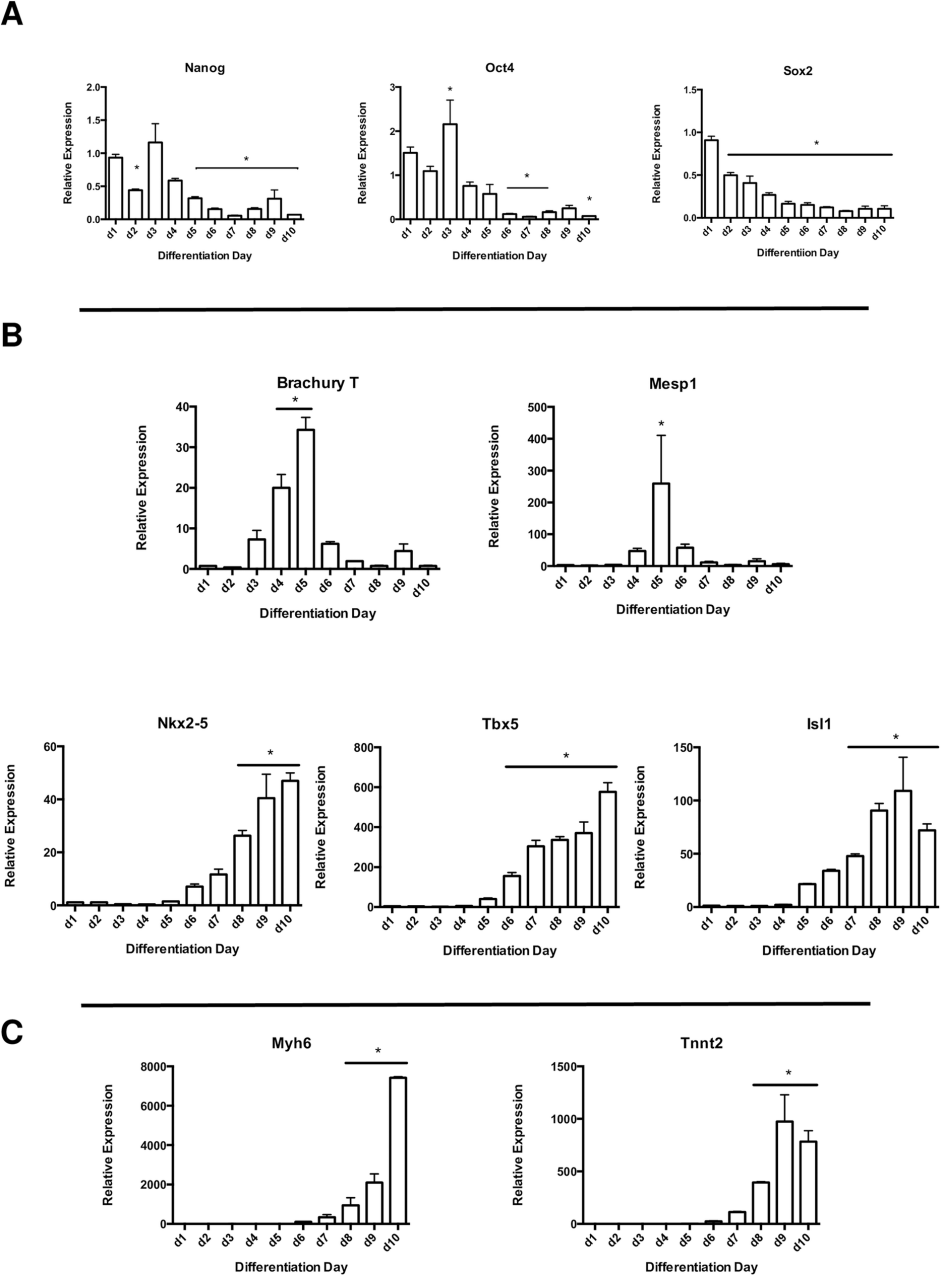
Gene expression dynamics for pluripotency-, mesoderm- and cardiac lineage-associated transcription factors in cardiac-differentiated Nkx2.5 EmGFP ES cells. **A)** pluripotency markers Nanog, Oct4 and Sox2, **B)** mesodermal marker Brachyury T and early cardiac lineage transcription factors Mesp1, Nkx2.5, Tbx5 and Isl1, **C)** cardiac-specific contractile proteins Myh6 and Tnnt2 (*p<0.05 vs. d0 undifferentiated; n = 3).

Gene expression analysis on non-sorted, total cell lysates was performed at each day of differentiation to confirm the loss of ES cell pluripotency and ensure cardiac-specific lineage commitment. Pluripotency genes including Nanog, Oct4 and Sox2 were expectedly reduced over time (**Fig 2A**), followed by a significant monophasic increase at d5 in the mesoderm lineage marker Brachury T as well as one of the earliest markers of cardiac development, Mesp1 (**Fig 2B**). Expression of transcription factors indicative of early cardiac lineage commitment such as Nkx2-5, Tbx5 and Isl1 all showed significant and progressive increases over time (**Fig 2B**). Notably, temporal expression of EmGFP appeared to correlate with that of Nkx2-5 (**Figs 1D and 2B**). Expression levels for functional genes associated with terminal cardiomyocyte differentiation such as Tnnt2 and Myh6 became significantly increased at d8 and displayed maximal expression at d9 and d10, respectively (**Fig 2C**). By comparing the various time points for live, EmGFP+ cell numbers with their associated transcriptional profiles, we selected d8 as the optimal window for CPC lncRNA analysis on the basis of relatively high CPC marker expression and total EmGFP+ cell numbers, as well as comparatively low expression of functional markers for terminal cardiomyocyte differentiation.

Nkx2-5 EmGFP cells were then differentiated to d8 and sorted via FACS (**S1A Fig**). Both EmGFP+ and EmGFP- cell fractions were collected, followed by RNA extraction. To verify enrichment of CPC-like cells in the EmGFP+ fraction, RT-PCR analysis was performed on the two fractions to compare expression of genes associated with pluripotency, early cardiac lineage commitment and terminal differentiation. When compared to undifferentiated ES cells, the EmGFP+ fraction displayed much higher levels of CPC-specific as well as lower levels of pluripotency-associated genes relative to the EmGFP- fraction (**S1B Fig**), indicating that the separation of differentiated Nkx2-5 EmGFP cells on the basis of EmGFP positivity was a reliable method to enrich for CPCs.

### CPC lncRNA analysis

Whole transcriptome sequencing was then performed to identify and assess both annotated and unannotated transcripts in the d8 EmGFP+ population–hereafter referred to as d8 CPCs. Additionally, a murine *ex vivo*-derived population of CPCs was also included in the sequencing analysis. These cells are a cardiosphere-derived Lin^-^c-kit^+^ (CLK) population of adult murine CPCs capable of both *in vitro* and *in vivo* differentiation into cardiomyocytes, vascular endothelial and smooth muscle cells [19, 26]. Use of these cells was predicated on the notion that the d8 CPCs represented an embryonic-in-origin, artificially-generated progenitor cell population whose transcriptional profile may not faithfully recapitulate that of either a true embryonic or adult CPC and that inclusion of CLK CPCs in the sequencing analysis would buttress against such misgivings in terms of data validity. Additionally, CLK CPCs were preselected for use in favor of another primary murine, cardiosphere-derived Sca1+ CPC population by virtue of their higher expression levels of mesodermal and cardiac lineage markers (**S2 Fig**). By performing comparative sequencing analysis on the d8 and CLK CPC populations, we hoped to uncover previously unidentified transcripts differentially expressed relative to undifferentiated ES cells which could in turn lend credence to their potential involvement in CPC development and/or homeostasis.

**Fig 3:**
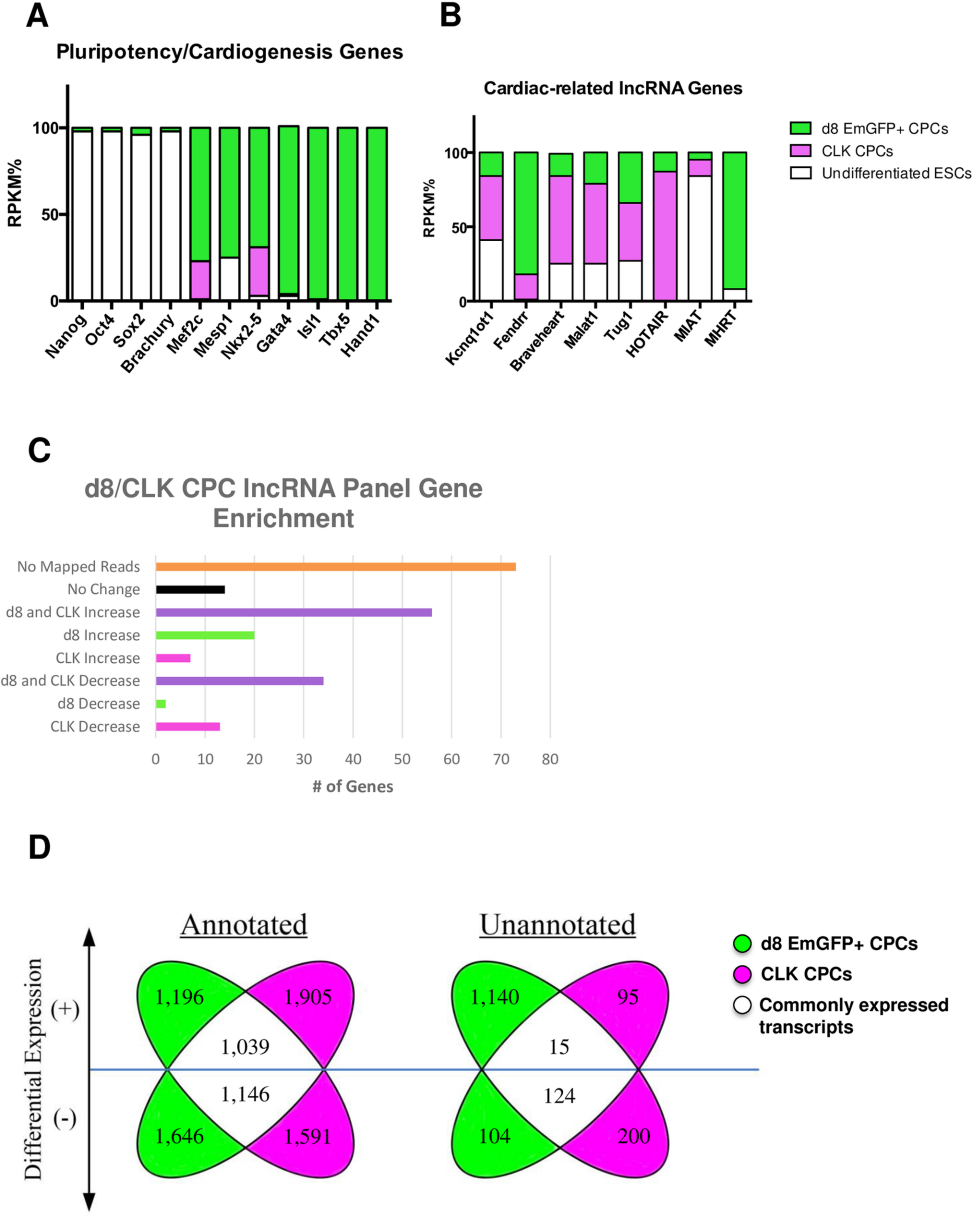
RNA-Seq data for d8 and CLK CPCs confirms enrichment for protein-coding and lncRNA genes associated with CPCs as well as the distribution of differentially-expressed annotated and unannotated genes. **A)** RNA-seq-derived RPKM values for pluripotency and cardiac-related genes as proportionally represented by d8/CLK CPCs and undifferentiated ES cells, **B)** distribution of RPKM values for d8/CLK CPCs and undifferentiated ES cells with respect to known cardiac-related lncRNAs, **C)** graphical summary of d8/CLK enrichment for panel of lncRNA genes, previously identified by Wamstead et al, in cardiac-differentiated ES cells, **D)** Biaxial Venn plot showing distribution of differentially-expressed annotated and unannotated genes in d8/CLK CPCs relative to undifferentiated ES cells, highlighting transcripts that are commonly up- and down-regulated.

Upon alignment and mapping, preliminary analysis of sequencing data obtained for both the d8 and CLK CPCs provided confirmation that these two populations were strongly representative of cardiac lineage type cells. As a percentage of total RPKM (*r*eads *p*er *k*ilobase per *m*illion mapped reads), pluripotency gene expression levels were significantly reduced in these two populations relative to undifferentiated ES cells, while hallmark cardiogenesis genes indicative of cardiac lineage commitment were highly enriched for (**Fig 3A**), particularly within d8 CPCs.

Further analysis of the annotated portion of the sequencing data–for which a total of 8,523 genes were determined to be differentially expressed (**Fig 3D**)–revealed an enrichment in both CPC populations for lncRNA genes previously implicated in cardiac development, homeostasis and/or disease [27], including Kcnq1ot1, Fendrr, Braveheart, Malat1, Tug1 and Hotair (**Fig 3B**). When cross-referenced to a panel of 195 known lncRNA transcripts identified in a screen for genes involved in directed cardiac differentiation using an alternative *in vitro* culturing system [28], a total of 108 of these transcripts (~55%) were determined to be differentially expressed in either or both of the d8 and CLK CPC samples relative to undifferentiated ES cells (**Fig 3C**), whereas the remaining 87 lncRNA genes included in this list either showed no relative expression changes or exhibited no mapped reads with respect to those genes in the d8 /CLK CPC samples.

The use of bioinformatics analysis tools that are tailored for screening sequencing data for identifiable features unique to lncRNA genes, such as PhyloCSF [29] and PORTRAIT [30], was beyond the scope of our expertise. Instead, we sought to identify putative lncRNA genes based on their lack of inclusion in the most current mouse genome build, mm10. That is, any unique transcripts that mapped to unannotated regions of the mouse genome were considered putative (though non-definitive) lncRNA gene candidates. We then compared the unannotated data sets from the d8 and CLK CPCs and looked for any commonly up- or down-regulated candidate genes–the operating presumption being that a transcript that is commonly expressed (or reduced in expression) in these two disparate-in-origin CPC populations would have a much higher likelihood of being biologically relevant.

**Fig 4:**
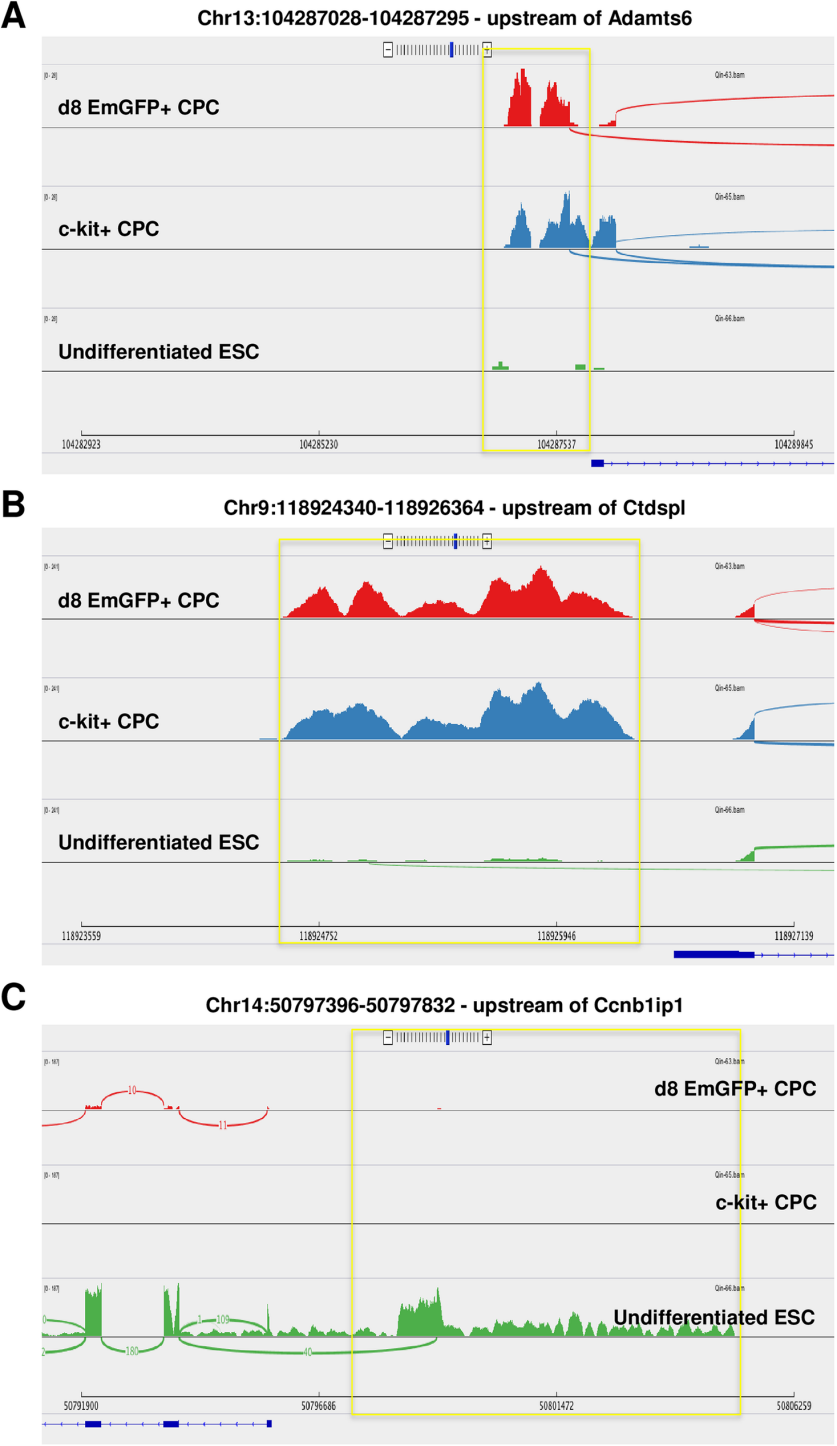
Sashimi read density plots show unannotated, putative lncRNAs common to d8/CLK CPCs that are differentially expressed relative to undifferentiated ES cells and map adjacent to cardiac-related protein-coding genes. **A)** 247nt transcript commonly upregulated in d8/CLK CPCs, approximately 1Kb upstream of Adamts6, B) 2,024nt transcript upregulated in d8/CLK CPCs, immediately upstream of Ctdspl, C) 1,378nt transcript downregulated in d8/CLK CPCs, upstream of Ccnb1ip1.

A total of 1,678 unannotated, putative lncRNA genes were identified and shown to be differentially expressed within d8 and CLK CPCs compared to undifferentiated ES cells (**Fig 3D**). Of these, 15 were commonly upregulated in both cell types while 124 were commonly downregulated. Notably, several of these transcripts mapped to regions of the mouse genome that were closely proximal or distal to protein-coding genes that are known to be involved in or related to cardiac development, homeostasis or disease. For example, a 247 nucleotide (nt) transcript 1Kb upstream of the transcription start site of Adamst6, a member of a thrombospondin-like metalloproteinase family implicated in Weill-Marchesani Syndrome (WMS–which features cardiac fibrosis as a component of its pathogenic sequelae) [31] and congenital heart disease (CHD) [32], was upregulated in both d8 and CLK CPCs and appeared to generally correlate positively with Adamts6 expression levels, as its absence in undifferentiated ES cells was also concomitant with a lack of Adamts6 expression (**Fig 4A**). As well, a 2,024nt transcript was mapped immediately upstream of Ctdspl, a phosphatase that, through Snail, enhances focal adhesion and cell migration [33] and also contains an intronic miRNA–miR26a –that directly inhibits expression of the cardiac transcription factor Gata4 [34] (**Fig 4B**). Similar to Adamts6, Ctdspl expression levels appeared to correlate positively with the emergent, elevated levels of this unannotated transcript in the d8/CLK CPCs relative to undifferentiated ES cells.

Additionally, of the transcripts that displayed reduced expression in the d8 and CLK CPCs, several also mapped to regions adjacent to cardiac-related protein-coding genes. One transcript mapped within 3Kb of the transcription start site for Ccnb1ip1 (**Fig 4C**), an E3 ubiquitin ligase that targets CyclinB1 for degradation and is upregulated when the cardiac transcription factor Hand2 is knocked down in cardiac neural crest-derived cells [35]. Interestingly, the Sashimi plot for this transcript also indicated that it may function as an alternative splice variant conjoined with the second exon of Ccnb1ip1.

## Discussion

Here, we generated an *in vitro*, ES cell-derived population of enriched Nkx2.5-expressing mouse CPCs and employed RNA-seq to profile lncRNAs that were either increased or decreased in these cells relative to undifferentiated ES cells in an effort to enumerate lncRNA species that are potentially implicated in or required for cardiac development. To complement this profiling, we also performed RNA-seq on an alternative population of adult, *ex vivo*-derived CLK CPCs. Several known cardiac-related lncRNAs were shown to be highly enriched for in these two CPC populations including Fendrr, Braveheart, Hotair, Tug1 and Malat1. In addition, when RNA-seq data from the two CPC populations was compared to a panel of 195 previously-identified lncRNAs implicated in a variant model of cardiac-directed, ES cell-based differentiation, over half (108) were determined to be differentially expressed when compared to undifferentiated ES cells. Taken together, these data suggest that these CPC populations can be used as valid tools and models for further investigating lncRNAs specifically involved in cardiac differentiation.

A more rigorous and comprehensive interrogation of the two CPC RNA-seq data sets was limited by a deficit in bioinformatics expertise in lncRNA profiling software such as PhyloCSF and PORTRAIT. In lieu of this, however, when screening on the basis of unannotated genes, we were able to identify a significant quantity of transcripts– 1,678 –that were expressed (or repressed) in CPCs relative to undifferentiated ES cells, some of which shared close proximity to cardiac-related protein-coding genes. Future efforts focused on utilizing lncRNA mining software for analyzing sequence information within these transcripts to screen for likely lncRNA candidate genes could potentially lead to the identification of novel molecular targets for improving our understanding of the molecular mechanisms that regulate CPC development and homeostasis. Such work will be crucial for advancing stem cell-based therapies as an effective treatment for CVD.

## Supporting information

S1 Fig Flow cytometry sorting and RT-PCR analysis of d8-differentiated Nkx2.5 EmGFP+ ES cells demonstrates enrichment for cardiac lineage-committed cells. **A)** Depiction of gating strategy for sorting Nkx2.5 EmGFP+/- ES cells, **B)** RT-PCR gene expression analysis in EmGFP+/- cells shows enrichment for non-pluripotent, cardiac lineage-committed cells.
(PDF)

S2 Fig Comparative RT-PCR analysis of cardiospehere-derived c-kit- and Sca1- CPCs shows CLK CPCs generally exhibit higher levels of CPC markers and lower levels of pluripotency genes. (PDF)

S1 Table Contained in the far left column of this spreadsheet is a list of 195 lncRNA genes identified previously by Wamstead et al. that were determined to be differentially expressed in an alternative model for *in vitro*, cardiac-directed ES cell differentiation. The adjacent right column indicates which, if either, of the d8 EmGFP+/CLK CPCs also exhibited differential expression in these lncRNA genes and, if so, in what directional capacity (i.e.–increased or decreased) relative to undifferentiated ES cells.
(XLSX)

S2 Table These worksheets contain all commonly expressed RNA-seq mapped genes, both annotated and unannotated, from d8 and CLK CPCs–with corresponding alignment track information–determined to be differentially expressed relative to undifferentiated ES cells. (XLSX)
