# Supplementary figures and images for: Characterization and analysis of long non-coding rna (lncRNA) in In Vitro- and Ex Vivo-derived cardiac progenitor cells
Source: PLoS One. 2017 Jun 22;12(6):e0180096. doi: 10.1371/journal.pone.0180096 (PMC5481004; doi:10.1371/journal.pone.0180096)

A

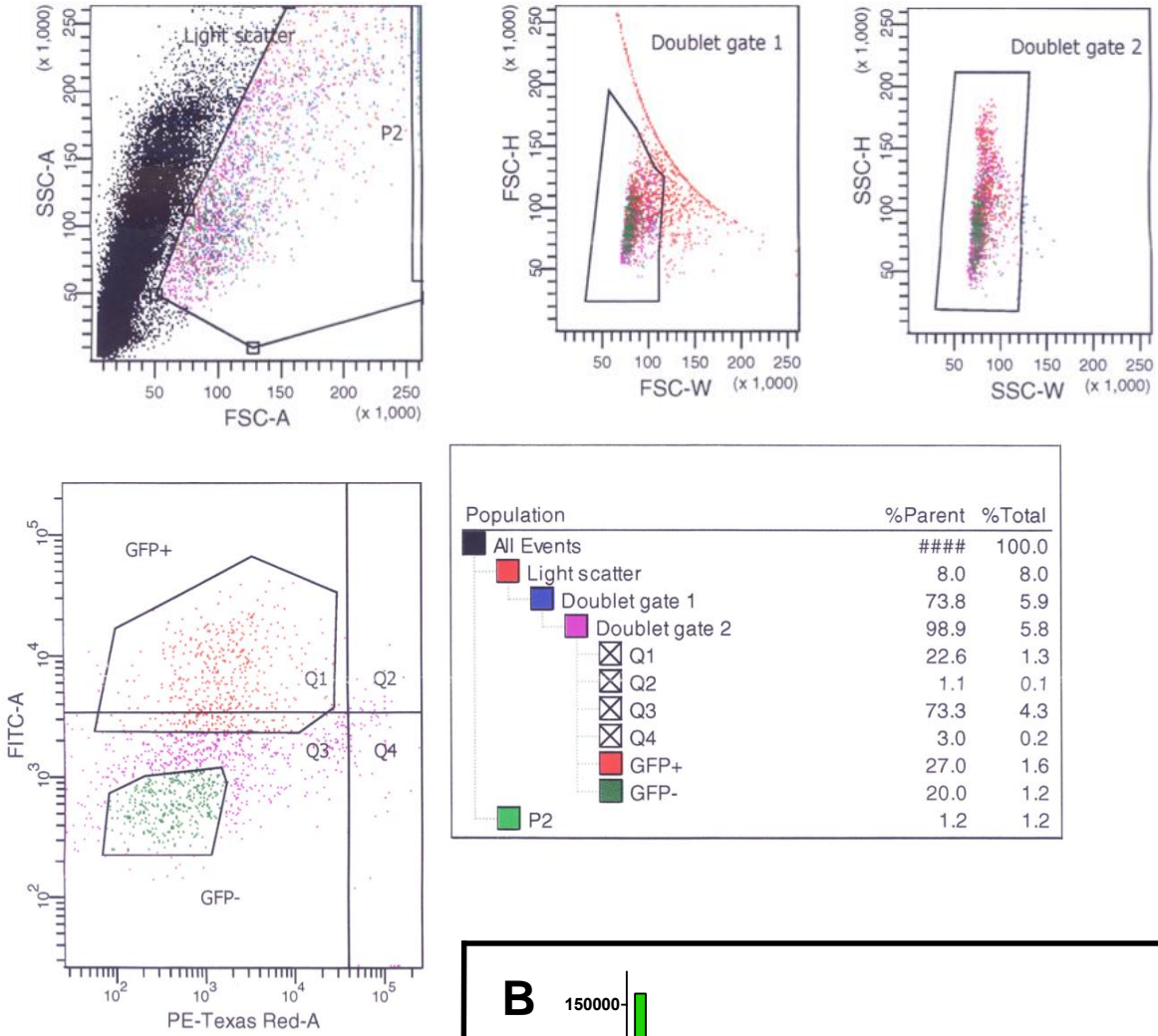

B

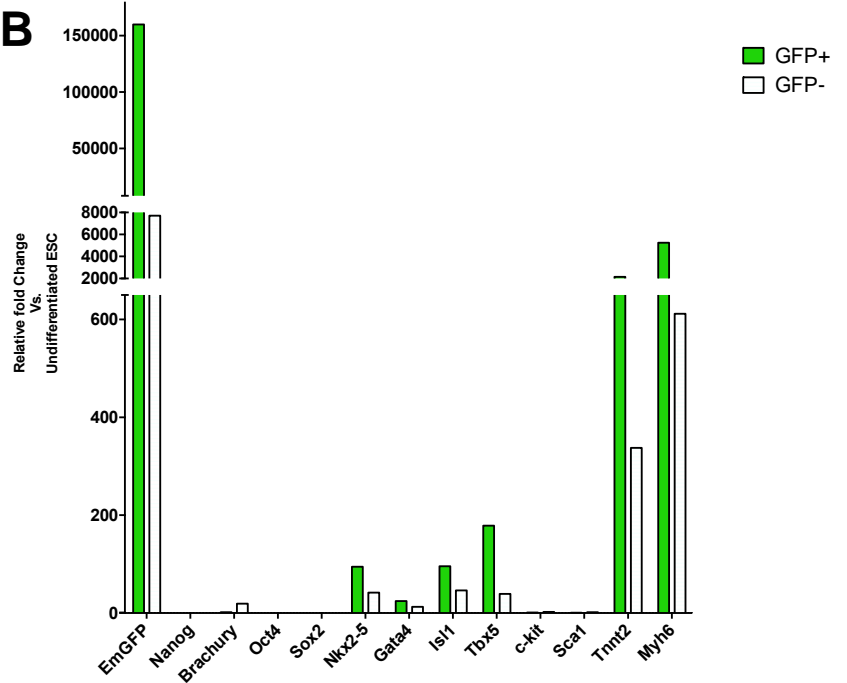

Supplement: S1 Fig — A) Depiction of gating strategy for sorting Nkx2.5 EmGFP+/- ES cells, B) RT-PCR gene expression analysis in EmGFP+/- cells shows enrichment for non-pluripotent, cardiac lineage-committed cells. (PDF) [file pone.0180096.s001.pdf]

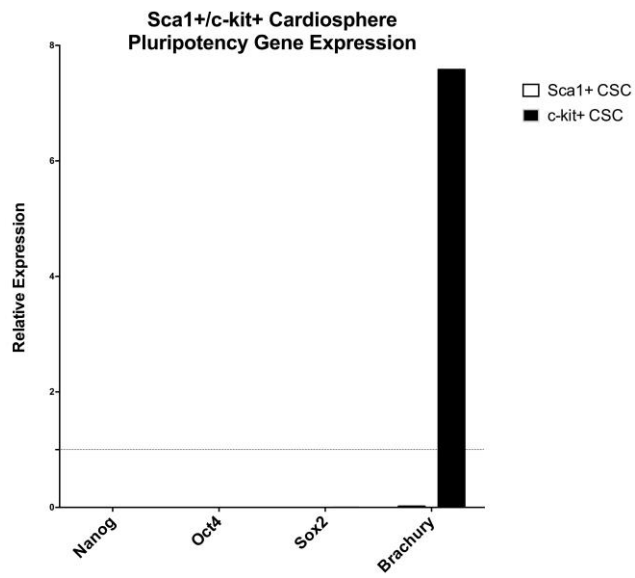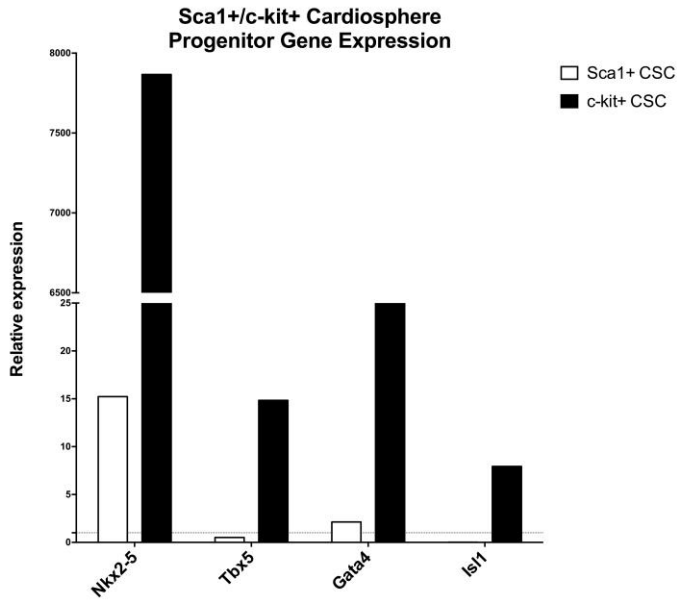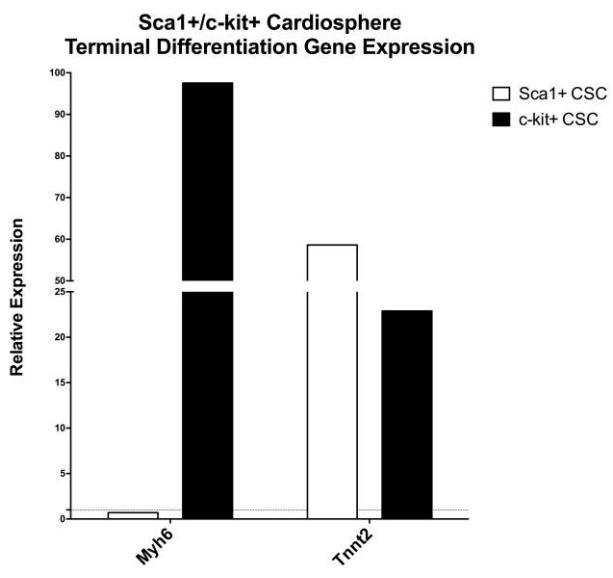

Supplement: S2 Fig — (PDF) [file pone.0180096.s002.pdf]
